# Antidiabetic Activity of *Pterospermum acerifolium* Flowers and Glucose Uptake Potential of Bioactive Fraction in L6 Muscle Cell Lines with Its HPLC Fingerprint

**DOI:** 10.1155/2014/459376

**Published:** 2014-10-21

**Authors:** Rathinavelusamy Paramaguru, Papiya Mitra Mazumder, Dinakar Sasmal, Venkatesan Jayaprakash

**Affiliations:** Department of Pharmaceutical Sciences, Birla Institute of Technology, Mesra, Ranchi, Jharkhand-835215, India

## Abstract

The present study was designed to estimate the detailed antidiabetic activity of* Pterospermum acerifolium* (L.) Willd flowers.* In vitro* alpha amylase inhibition study was carried out on 50% ethanol extract of flowers (PAFEE) and its various fractions. The active ethyl acetate fraction (PAFEF) was subfractionated into three subfractions (PAFE1, PAFE2, and PAFE3) and subjected to acute toxicity studies followed by antidiabetic screening* in vivo* by streptozotocin-nicotinamide induced type II diabetes. Diabetic animals treated with PAFE2 (30 mg/kg) reduced the levels of fasting blood glucose, significantly (*P* < 0.001) compared to that of diabetic control animals. Histological studies on drug treated groups did not show remarkable positive changes in *β*-cells. PAFE2 showed 32.6 ± 1.93% glucose uptake over control and, in the presence of PI3K inhibitor wortmannin, declined to 13.7 ± 2.51%. HPLC analysis of PAFE2 reveals the presence of quercetin and apigenin as major constituents and both are inhibiting the glycogen phosphorylase enzyme in molecular modelling studies. The study evidenced strongly that the probable glucose lowering mechanism of action of active subfraction PAFE2 is by increasing the glucose uptake in peripheral tissues and by inhibition of gluconeogenesis.

## 1. Introduction

Diabetes mellitus is the most common endocrine disorder resulting from insulin deficiency which in turn leads to chronic hyperglycemia with disturbances of carbohydrate, fat, and protein metabolism [1]. It is majorly classified into insulin dependent diabetes mellitus (Type I) and noninsulin dependent diabetes mellitus (Type II); type II diabetes mellitus is the most common endocrine disorder worldwide, covering about 90–95% of all diabetes cases [2]. The classification and pathogenesis of type II diabetes involves abnormalities in glucose and lipid metabolism, inadequate insulin secretion from pancreatic beta-cells, and resistance to insulin activity [3]. The prevalence of type 2 diabetes continues to increase at an alarming rate globally. Even though the pathogenesis and long term complications of type 2 diabetes are fairly well known, its treatment has remained challenging [4]. Currently available therapeutic measurements to treat type II diabetes mellitus also have certain adverse effects like causing hypoglycemia at higher doses, liver problems, lactic acidosis, and diarrhea [5].

*Pterospermum acerifolium* (L.) Willd (Sterculiaceae) is commonly known as Kanakchampa, traditionally used for hemostasis, inflammation, ear pain, stomach-ache, blood troubles, small pox, leucorrhoea, leprosy, ulcer, and tumours and as antihyperglycemic agent, laxative, and anthelmintic [6, 7]. The flowers of* Pterospermum acerifolium *are commonly consumed by the tribal folks of Chota Nagpur region of Jharkhand, India. The phytoconstituents reported from flower were 24 *β*-ethylcholest-5-en-3-beta-0-alpha-cellobiside, 3,7-diethyl-7-methyl-1 : 5-pentacosanolide, n-hexacosane-1,26-dioldilignocerate, friedelan-3 *α*-ol, and its *β*-isomer, *β*-amyrin, *β*-sitosterol, n-triacontanol, n-hexacosane-1,26-diol, myristic, palmitic, stearic, arachidic, behenic, lignoceric, oleic, linoleic, linolenic acids, kaempferol, 4′ methoxy-kaempferol, and kaempferide-7-O-*β*-D-glucopyranoside [8]. Literature survey of plant showed that these plant extracts are showing antidiabetic properties but knowledge is very imprecise. The studies if at all are limited to extract level only and moreover detailed parameters, which could be claimed as well defined hypoglycemic activity of the plant is lacking. In order to explore the full potential of this plant as useful antidiabetic agent, it is necessary to carryout systemic studies of these plants and extracts responsible for antidiabetic activity. Hence this work is designed for evaluation of the antidiabetic property on the basis of bioactivity guided fractionation, estimation of mechanism of action of bioactive subfraction by which it controls the blood glucose level, and identification of bioactive constituents present in the active subfraction by HPLC analysis.

## 2. Materials and Methods

### 2.1. Plant Material

Flowers of* Pterospermum acerifolium* collected from the campus of BIT Mesra, Ranchi in the month of March 2011. The plant material had been identified and authenticated from Dr. Karthikeyan, taxonomy department of Botanical survey of India (BSI), Kolkata. The voucher specimen (CNH/48/2012/Tech II/805) was retained in the Department of Pharmaceutical Sciences, BIT Mesra, Ranchi for future reference.

### 2.2. Extraction and Fractionation

The shade dried flowers were powdered (1 kg) and extracted with 50% ethanol for the period of 72 h and subsequently concentrated to obtain a yield of 61.32 g of 50% ethanol extract of flower (PAFEE). Twenty-five grams of the extract was dissolved in distilled water and extracted successively with hexane (PAFHF), chloroform (PAFCF), and ethyl acetate (PAFEF) and concentrated to dryness to obtain the respective fractions.

### 2.3. *α*-Amylase Inhibition Assay

A modified form of Sigma-Aldrich method was used [9]. The *α*-amylase inhibition was expressed as percent of inhibition and it was calculated as 100 − % reaction where by the % reaction = (maltose in test/maltose in control) × 100.

### 2.4. Subfractionation of Ethyl Acetate Fraction

The ethyl acetate fraction was subfractionated by column chromatography. Crude fractions were adsorbed in silica gel (mesh 60–120) for uniform mixing. Using dichloromethane as solvent, the column was packed with silicagel (mesh 100–200). The column was eluted with dichloromethane followed by methanol, dichloromethane in the regular increased proportion of methanol. Each fraction was tested in TLC plate for its homogeneity and pulled together into three fractions and evaporated under room temperature to yield the subfractions PAFE1, PAFE2, and PAFE3.

### 2.5. Animals

Healthy male albino rats (Wistar strain 180–200 g) were procured from the animal house of Birla Institute of Technology. They were housed in clean polypropylene cages with free access to standard laboratory pellet diet and water at room temperature 27 ± 2°C with an inverted 12 : 12 h light-dark cycle and relative humidity of 60%. The animals were acclimatized for 7 days before the commencement of the experiments. The experimental protocols were approved by the Institutional Animal Ethics Committee (BIT/PH/IAEC/34/2011).

### 2.6. Acute Oral Toxicity Studies and Dose Fixation

Toxicity studies conducted on female mice based on OECD guidelines 423 for acute oral toxicity-acute toxic class method. For ethyl acetate fraction (PAFEF) 2000 mg/kg was fixed as initial dose and for subfractions (PAFE1, PAFE2, and PAFE3) 300 mg/kg was fixed as initial dose and the test substance was administered in a single dose by using an intragastric tube. Animals were observed individually after dosing at least once during the first 30 minutes, periodically during the first 24 hours, with special attention given during the first 4 h, and daily thereafter, for a total of 14 days.

### 2.7. Induction of Diabetes

Streptozotocin (STZ) was dissolved in citrate buffer (pH 4.5) and nicotinamide was dissolved in normal physiological saline. Noninsulin dependent diabetes mellitus was induced in overnight fasted rats by a single intraperitoneal injection of STZ (60 mg/kg b.w.). 15 min later, the rats were given the intraperitoneal administration of nicotinamide (120 mg/kg b.w.). Hyperglycemia was confirmed by the elevated glucose levels, determined at 72 h. The animals with blood glucose concentration more than 250 mg/dL were included in the study [10].

### 2.8. Experimental Design

Animals were divided into 11 groups and each group consisted of 6 animals.

 Group I: normal control (2% tween 80; 1 mL/kg b.w.).
 Group II: diabetic control rats receiving sterile water.
 Group III: PAFEF1; diabetic animals treated with PAFEF 200 mg/kg b.w.
 Group IV: PAFEF2; diabetic animals treated with PAFEF 400 mg/kg b.w.
 Group V: PAFE1-1; diabetic animals treated with PAFE1 15 mg/kg b.w.
 Group VI: PAFE1-2; diabetic animals treated with PAFE1 30 mg/kg b.w.
 Group VII: PAFE2-1; diabetic animals treated with PAFE2 15 mg/kg b.w.
 Group VIII: PAFE2-2; diabetic animals treated with PAFE2 30 mg/kg b.w.
 Group IX: PAFE3-1; diabetic animals treated with PAFE3 15 mg/kg b.w.
 Group X: PAFE3-2; diabetic animals treated with PAFE3 30 mg/kg b.w.
 Group XI: STD drug; diabetic animals treated with 600 *μ*g/kg b.w. of glibenclamide.

Plant extracts, glibenclamide and vehicle, were administered via the intragastric tube for 30 days.

### 2.9. Biochemical Analysis

Oral glucose tolerance test (OGTT) was performed in overnight fasted normal and drug treated rats on 15th day of treatment [11]. The fasting blood glucose was measured on day 0, 14, 21, and 28 by using glucometer (Bayer's blood glucose measuring kit with counter strips). On the 30th day, blood was collected in the EDTA coated tubes for measuring glycosylated hemoglobin (HbA1_C_) by using commercial kit (coral HbA1_C_ estimation kit) and blood was collected in tubes without anticoagulant for serum preparations. Serum was analyzed for ALT (Alkaline transaminase), AST (Aspartate transaminase), ALP (Alkaline phosphatase) by cogent Serum-ALT, AST, ALP Estimation Kits (Span diagnostics limited). Triglycerides, Total cholesterol (TC), HDL-C was estimated by Autospan triglycerides, TC, HDL-C estimation kits (Span diagnostics limited). Further TBARS [12], catalase [13], superoxide dismutase [14], and reduced glutathione [15] were estimated from liver homogenate.

### 2.10. Histopathology of Pancreas

The pancreas from normal control, diabetic control, and maximum drug dose treated animals were blotted free of mucus. The tissues were washed in normal saline, cut into desired size, and fixed in 10% formalin for 24 h. After fixation, tissues were cleaned and embedded in paraffin. Sections of tissue were made in rotary microtome of 5 *μ*m in thickness and mounted on slides. The mounted slides were stained with haematoxylin and eosin for photographic observations.

### 2.11. Glucose Uptake Study on L6 Muscle Cell Lines in the Presence and Absence of Wortmannin

The determination of cell viability was performed by MTT assay [16]. Twenty four hour cell cultures with 70–80% confluency in 40 mm petri plates were allowed to differentiate by maintaining in DMEM with 2% FBS for 4–6 days. The differentiated cells were used to measure the cell-associated glucose using glucose assay kit (Biovision Inc., USA) [17]. For PI3-K inhibition studies, L6 myotubes were treated with wortmannin 100 nM, 30 min prior to the incubation with the PAFE2 (1000 *μ*g/mL) and standard Insulin (1 IU/mL) followed by the glucose uptake assay [18].

### 2.12. HPLC Analysis

The active subfraction was subjected to analysis by being dissolved in HPLC grade methanol (1 mg/mL). The HPLC system (Waters 510, Japan) was equipped with dual pump binary system, C_18_ reversed-phase column, GL Sciences Inc., Japan (I.D. 4.6 mm × 250 mm, 5 *μ*m). Flow rate and injection volume were 1.0 mL/minute and 20 *μ*L, respectively. The samples were monitored with UV detection at 320 nm at room temperature. Separation was achieved with a two-pump linear gradient program for pump A (water containing 0.1% formic acid) and pump B (acetonitrile). Elution was started with a gradient of 10% B changing to 70% in 25 minutes and finally to 10% in 35 minutes followed by washing for 40 minutes. The chromatographic peaks of the analytes were confirmed by comparing their retention time and UV spectra with corresponding reference standards. The reference standards used were gallic acid, chlorogenic acid, rutin, quercetin, apigenin, and kaempferol.

### 2.13. Molecular Docking Studies

Quercetin and apigenin were identified as the major components present in bioactive subfraction. Hence both were selected as a ligand for molecular docking studies as remaining compounds were not of any therapeutic significance.

The autodock 4.2 docking software was used to perform molecular docking simulation between rabbit muscle glycogen phosphorylase and quercetin, apigenin. The crystal structure of glycogen phosphorylase complexed with acyl urea derivatives (PDB CODE: 1WV1) at 2.26 Å resolution was downloaded from the RCSB Protein Data Bank (http://www.rcsb.org/pdb/home/home.do). MGLTools-1.4.6 was used to prepare protein (protein.pdbqt) and to write grid parameter file (protein.gpf) and docking parameter file (ligand.dpf). Protein preparation includes (i) removal of water and ions and extraction of cocrystallized ligand; (ii) addition of polar hydrogens; (iii) assignment of AD4 atom type; and finally (iv) assignment of Gasteiger charges. The grid maps representing the native ligand in the actual docking target site were calculated with autogrid 4 with box dimension of 60 × 60 × 60 Å and spacing of 0.375 Å by taking centre of the ligand as centre of the grid. Docking of ligand was done with default parameters except keeping 50 runs.

### 2.14. Statistical Analysis

The results are expressed as mean ± standard error of the mean (SEM). Statistical analysis of all the data were evaluated according to one-way analysis of variance (ANOVA) using statistical software Graphpad prism version 6. The significance of difference was evaluated using one-way ANOVA followed by Dunnett's multiple comparison test. Probability values of ^aaaa^ *P* < 0.0001, ^aaa^ *P* < 0.001, ^aa^ *P* < 0.01, ^a^ *P* < 0.05 were compared with normal control. Probability values of ^bbbb^ *P* < 0.0001, ^bbb^ *P* < 0.001, ^bb^ *P* < 0.01, ^b^ *P* < 0.05 were compared with disease control.

## 3. Results

### 3.1. *In Vitro* *α*-Amylase Inhibition Study

The results of *α*-amylase inhibition study revealed that ethyl acetate fraction (PAFEF) possess higher enzyme inhibition potential (73.70%) compared to all other fractions followed by 50% ethanolic extract (PAFEE) which showed noticeable enzyme inhibition potential (47.44%); next to that, chloroform fraction showed 21.24% inhibition potential and n-hexane fraction did not show positive inhibition potential. Hence for further studies ethyl acetate fraction (PAFEF) was selected.

### 3.2. Acute oral Toxicity

Acute oral toxicity studies revealed the nontoxic nature of flowers of* P. acerifolium* at the dose levels tested. No lethality or toxic reactions were observed until the end of the study. Mortality was not recorded during 14 days on drug treated animals. Hence the doses were fixed as 200 mg/kg and 400 mg/kg for PAFEF and 15 mg/kg and 30 mg/kg for subfractions (PAFE1, PAFE2, and PAFE3).

### 3.3. Effect of* P. acerifolium* on OGTT

In oral glucose tolerance test, the blood glucose levels of glucose loaded experimental animals were increased markedly at 30 min. PAFE2-2 (30 mg/kg) inhibited the increasing blood sugar level significantly (*P* < 0.01) in the 60th min and the 120th min when compared with the diabetic control. None of the other fractions controlled the blood glucose significantly when compared with the diabetic control (Table 1).

### 3.4. Effect of* P. acerifolium* on Fasting Blood Glucose Levels, Body Weight, and HbA1_C_

In flowers, fasting blood glucose levels were increased significantly (*P* < 0.0001) compared with the normal control in all the observed days. On day 14, when compared to diabetic control, PAFE2-2 (30 mg/kg) reduced the blood glucose level significantly (*P* < 0.01) in diabetic animals. On day 21, when compared to diabetic control, PAFE2-2 (30 mg/kg) reduced the blood glucose level significantly (*P* < 0.001), and PAFE1-1 (15 mg/kg) also reduced the blood glucose level significantly (*P* < 0.05). On day 28, when compared to diabetic control again, PAFE2-2 (30 mg/kg) reduced the blood glucose level significantly (*P* < 0.01). PAFE2-1 (15 mg/kg) also reduced the blood glucose level significantly (*P* < 0.05) (Table 2).

Body weight of the diabetic control animals reduced markedly compared to that of normal animals; PAFE2 (30 mg/kg) showed remarkable increase in body weight compared to diabetic control animals (Table 3). The levels of Glycosylated Hemoglobin (HbA1_C_) of diabetic control animals were increased significantly (*P* < 0.0001) compared to that of normal animals; PAFE2-2 (30 mg/kg) decreased the level of HbA1_C_ significantly (*P* < 0.001) compared to diabetic control (Table 3).

### 3.5. Effect of* P. acerifolium* on Levels of Serum Liver Enzymes and Lipid Parameters

Interpretation on the levels of ALT, AST, and ALP were found to be significantly increased in diabetic control animals (*P* < 0.0001) compared to that of normal animals. Diabetic animals treated with PAFE2-2 (30 mg/kg) showed the significant (*P* < 0.01) reduction on levels of ALT and AST when compared to the diabetic control animals and the identical one showed the significant reduction (*P* < 0.05) on level of ALP when compared to the diabetic control animals (Table 4).

Levels of triglycerides and total cholesterol of diabetic control rats were increased significantly (*P* < 0.0001) compared to that of normal vehicle treated animals and the level of HDL-C (*P* < 0.0001) on the same animals was decreased significantly compared to that of normal vehicle treated animals. Treatment with PAFE2-2 (30 mg/kg) decreased the level of triglycerides (*P* < 0.001) and total cholesterol (*P* < 0.01) and increased the level of HDL-C (*P* < 0.001) significantly when compared to that of diabetic control. PAFE2-1 (15 mg/kg) decreased the level of triglycerides (*P* < 0.01) and total cholesterol (*P* < 0.05) and increased the level of HDL-C (*P* < 0.05) significantly (Table 5).

### 3.6. Effect of* P. acerifolium* on* In Vivo* Antioxidant Enzymes Status

The level of thiobarbituric acid reactive substances (TBARS) was found to increase significantly (*P* < 0.0001) in diabetic control animals compared to that of normal vehicle treated animals and the levels of antioxidant enzymes such as catalase, SOD, and GSH were found to decrease significantly (*P* < 0.0001) in diabetic control animals compared to that of normal group. Diabetic animals treated with PAFE2-2 (30 mg/kg) showed the significant reduction (*P* < 0.01) on levels of TBARS compared to the diabetic control and increased the levels of catalase (*P* < 0.01) and SOD (*P* < 0.01) and reduced GSH (*P* < 0.01) significantly compared to that of diabetic control (Table 6).

### 3.7. Histopathological Studies

In diabetic control rats, the microscopic section of pancreas showed the features of insulitis and marked evidence of degeneration of *β*-cells revealing cellular swelling and cytolysis with pyknotic and fragmented nuclei. As compared to the diabetic control, animals treated with the ethyl acetate fraction and sub fractions of* Pterospermum acerifolium* did not showed any notable changes in islet cells granulation and cellular arrangements (data not shown).

### 3.8. Cell Viability and Glucose Uptake Assay

**Figure 1:**
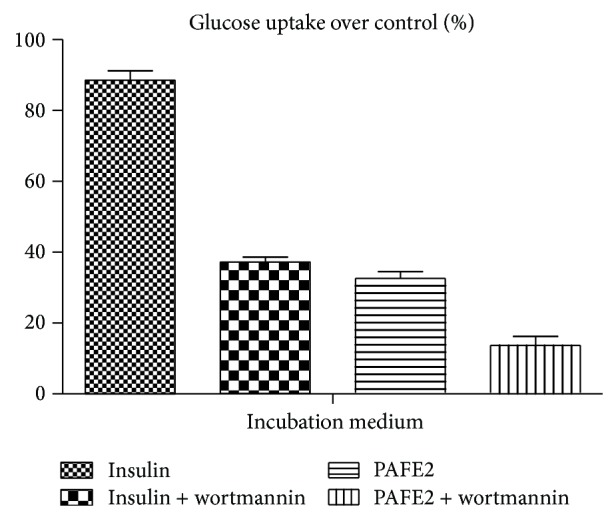
Effect of PAFE2 (1000 *μ*g/mL) and insulin (1 IU/mL) on glucose uptake in L6 muscle cell lines in the absence and presence of wortmannin (100 nM).

PAFE2 was evaluated for its cytotoxic activity by MTT assay. CTC_50_ value of PAFE2 were found to be >1000.00 *μ*g/mL. In Glucose uptake assay PAFE2 showed 32.6 ± 1.93% glucose uptake over control compared with the standard insulin (1 IU/mL) which showed 88.5 ± 2.69% glucose uptake over control. In the presence of PI3K inhibitor wortmannin, PAFE2 showed 13.7 ± 2.51% glucose uptake over control and the standard insulin (1 IU/mL) showed 37.2 ± 1.44% glucose uptake over control (Figure 1).

### 3.9. HPLC Fingerprint Analysis

**Figure 2:**
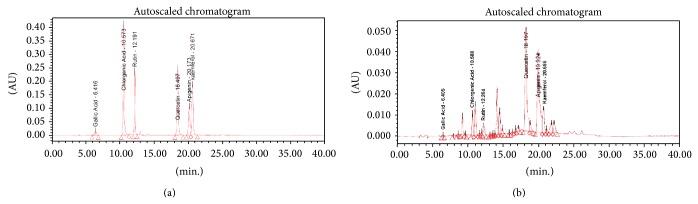
(a) HPLC chromatogram of reference standards (b) HPLC chromatogram of subfraction PAFE2.

The fingerprint analysis of active ethyl acetate subfraction PAFE2 of flowers of* Pterospermum acerifolium *showed the presence of gallic acid, chlorogenic acid, rutin, quercetin, apigenin, and kaempferol by comparing its retention time and UV spectrum with the reference standards (Table 7; Figure 2). Based on the peak area, quercetin and apigenin were found as the major constituents in PAFE2.

### 3.10. Molecular Modelling Studies

**Figure 3:**
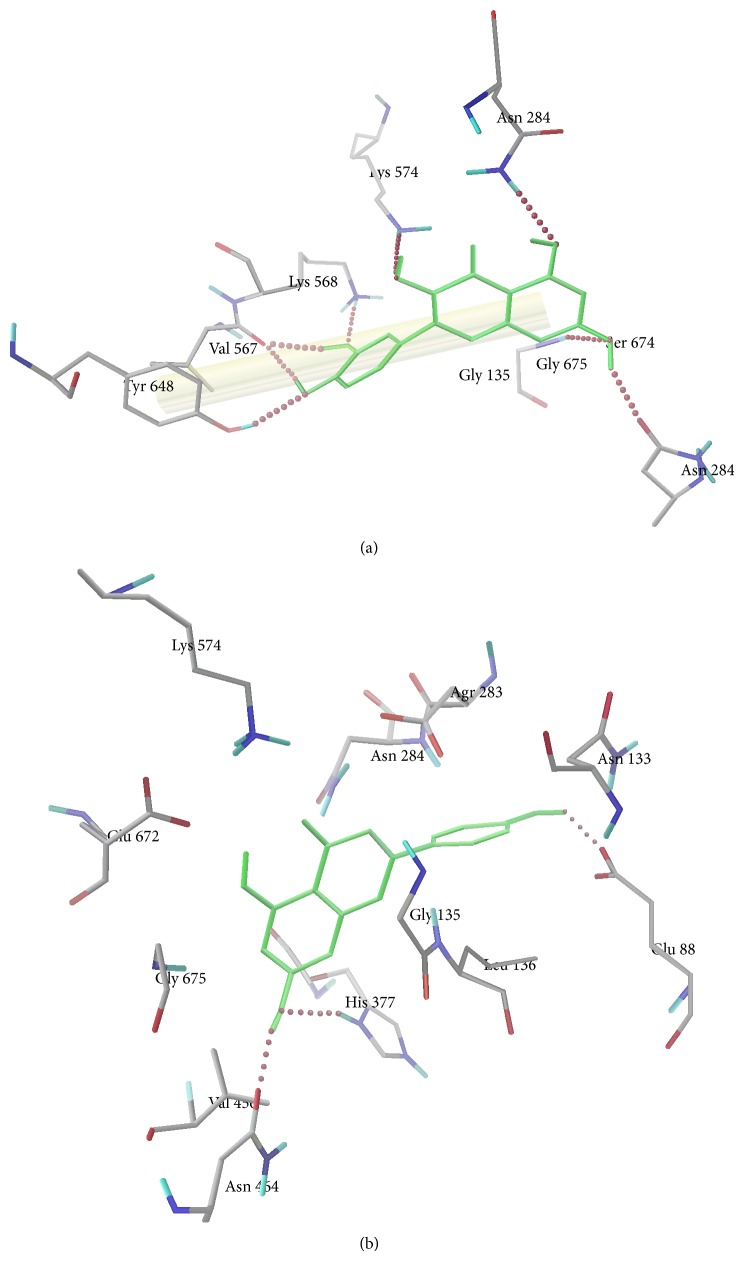
Hydrogen bond interactions between amino acid residues in the active site pocket of glycogen phosphorylase and (a) quercetin and (b) apigenin.

To investigate the interaction of quercetin and apigenin, molecular docking simulations of the binding of those molecules with glycogen phosphorylase active site were carried out using Autodock 4.2. From this study we found that quercetin is making eight H-bond interactions with the active site residues Asn 284, Lys 568, Lys 574, Tyr 648, Gly 675, Asn 484, and Val 567 and *π* electrons of flavone nucleus of quercetin is making *π*-*π* stacking interaction with Tyr 648 (Figure 3(a)). The estimated free energy binding of quercetin was found to be −8.27 kcal mol^−1^ with estimated inhibition constant (*K*_*i*_) of 0.87 *μ*M. Similarly, apigenin is making three hydrogen bond interactions with the active site residues Glu 88, His 377, and Asn 484 (Figure 3(b)). The estimated free energy binding of apigenin was found to be −8.08 kcal mol^−1^ with estimated inhibition constant (*K*_*i*_) of 1.2 *μ*M, which infers clearly that the major constituents of bioactive subfraction can inhibit glycogen phosphorylase enzyme.

## 4. Discussion

Diabetes mellitus is regarded as a noncurable but controllable disease. Progressive nature of the disease necessitates constant reassessment of glycaemic control in people with diabetes and appropriate adjustment of therapeutic regimens [19]. *α*-amylase catalyzes the hydrolysis of *α*-1,4-glucosidic linkages of starch, glycogen, and various oligosaccharides and simplifies the availability of sugars for the intestinal absorption. Inhibition of this enzyme activity in the digestive tract of humans is considered to be effective to control diabetes by diminishing the absorption of glucose decomposed from starch by this enzyme [20, 21]. In the present study, ethyl acetate fraction PAFEF was found as the potential *α*-amylase inhibitor amongst the tested extract and fractions. Hence PAFEF was selected for further subfractionation and* in vivo* studies.

Diabetic patients however cannot control their postprandial blood glucose efficiently due to the insufficient insulin secretion or response, which results in postprandial hyperglycemia and it is an important contributing factor for diabetic complications [22]. PAFE2 (30 mg/kg) treated diabetic rats significantly reduces the blood glucose levels at 60th and 120th min when compared to that of diabetic control and it implies the further scope of the drug to be studied in detail on diabetic complications using animal models.

The diabetic syndrome in rats administered STZ and partially protected with suitable dosages of nicotinamide is characterized by stable moderate hyperglycemia [23]. In the present study STZ-nicotinamide induced diabetic rats treated with PAFE2 (30 mg/kg) reduces the blood glucose level at 14th, 21st, and 28th days significantly and it showed clearly its antihyperglycemic effect. In diabetes, there is an increased glycosylation of a number of proteins therefore measurement of HbA1_C_ has proven to be particularly useful in monitoring the effectiveness of therapy [3]. Diabetic animals treated with PAFE2 (15 mg/kg & 30 mg/kg) reduced the HbA1_C_ levels significantly compared to that of diabetic control group and it could also be due to its antihyperglycemic property.

The level of activities of liver biomarker enzymes such as ALT, AST, and ALP were used in the evaluation of hepatic disorders and increase in the serum levels of these enzymes in diabetic rats that substantiated the hepatic damage [11]. The observed significant reduction in the serum level of these enzymes in PAFE2 (30 mg/kg and 15 mg/kg) treated diabetic animals indicates its hepatoprotective role in preventing diabetic complications. The elevation of serum triglycerides, total cholesterol, and the decline in the level of HDL-C was well documented in diabetic animals. In PAFE2 (30 mg/kg and 15 mg/kg) treated diabetic animals showed significant reduction in the level of triglycerides, total cholesterol, and raise in the level of HDL-C and it clearly showed its effect on lipid regulation systems.

In diabetic control animals it was found clearly that there was a marked increase in the levels of TBARS and decrease in the levels of antioxidant enzymes such as catalase, GSH, and SOD [24, 25]. Treatment with PAFE2 (30 mg/kg & 15 mg/kg) reduces the TBARS levels and increases the levels of antioxidant enzymes compared to that of diabetic control and it clearly implies its role against oxidative stress.

Histopathological studies showed that there was no regeneration of islet cells or no insulinogenic property and it reveals that the PAFE2 site of action may be extrapancreatic and not the regeneration of *β*-cells and the decrease in blood glucose may be attributed to the stimulation of glucose uptake by peripheral tissues and decrease in the gluconeogenesis. There are marked numbers of medicinal plants reported for antidiabetic activity without the stimulation of insulin secretion [23].

L6 myotubes is a well-established skeletal muscle model for studying glucose uptake process and it is one of the key insulin targeted tissues in maintaining whole body glucose homeostasis, through the stimulation of glucose uptake mediated by GLUT4 translocation [26]. The results obtained demonstrated that PAFE2 enhances the basal glucose uptake and it is clear that mimic insulin action. Many reports emphasize that PI3K plays a major role in insulin signaling pathway and regulates insulin-mediated glucose transport [27]. Pretreatment with specific PI3K inhibitor (wortmannin) resulted in a decline in the glucose uptake activity of PAFE2, suggesting that glucose transport is dependent primarily on the involvement of PI3K. Hence PAFE2 decreases blood glucose level by increasing the glucose uptake in peripheral tissues through PI3K mediated Glut-4 translocation. The HPLC fingerprint analysis reveals the presence of quercetin and apigenin as the major constituents. As quercetin and apigenin are the major components from the active subfraction PAFE2, they play a major role in establishing the mechanism of action for its antidiabetic activity. In the literature, it has been reported earlier that these compounds are well-known antidiabetic compounds in particular for its insulin mimetic property by increasing the glucose uptake in peripheral tissues [28]. The inhibition of hepatic glycogen phosphorylase could suppress glucose production arising from both glycogenolysis and gluconeogenesis [29]. Molecular modelling studies clearly revealed the inhibition potential of the identified major constituents quercetin and apigenin of PAFE2 by its lowest binding energy and estimated inhibition constant (*K*_*i*_) values. Hence the above results evidenced strongly that the probable glucose lowering mechanism of action of active subfraction PAFE2 is by increasing the glucose uptake in peripheral tissues and by inhibition of gluconeogenesis. Establishment of mechanism of action for antidiabetic property and identification of bioactive molecules responsible for the glucose lowering effect were undertaken for the first time with flowers of* Pterospermum acerifolium*.

## Figures and Tables

**Table 1:** Effect of ethyl acetate fraction and subfractions of flowers of *Pterospermum acerifolium* (L.) Willd on changes in blood glucose level during OGTT.

Treatment	Blood glucose level (mg/dL)			
	0th min	30th min	60th min	120th min
Normal control	75.2 ± 0.477	144.2 ± 0.477	139.2 ± 1.046	121.2 ± 1.600
Diabetic control	353 ± 11.8^aaaa^	442.8 ± 16.22^aaaa^	439.7 ± 18.97^aaaa^	398.2 ± 12.96^aaaa^
PAFEF1	234.6 ± 5.02	301 ± 5.373	278.8 ± 6.582	266.5 ± 7.602
PAFEF2	233 ± 16.8	292.2 ± 16.11	262.7 ± 15.95	249 ± 16.48
PAFE1-1	226 ± 7.44	278.8 ± 6.258	260.2 ± 8.845	246.5 ± 7.610
PAFE1-2	235 ± 13.5	285.7 ± 15.82	260.7 ± 15.68	249.7 ± 14.71
PAFE2-1	221 ± 6.11	275 ± 8.418	253.3 ± 7.032	236.5 ± 9.021
PAFE2-2	194 ± 7.77^bb^	247.7 ± 6.556^bbb^	221.3 ± 5.760^bb^	200 ± 7.554^bb^
PAFE3-1	269 ± 11.3^aa^	318.5 ± 15.64^aa^	313.3 ± 12.30^aaa^	309.3 ± 14.18^aaa^
PAFE3-2	264 ± 9.77^aa^	314.8 ± 9.365^a^	306 ± 10.17^aa^	295.8 ± 9.203^aa^
STD drug	176 ± 6.90^bbb^	225 ± 4.892^bbbb^	193 ± 4.235^bbb^	177.3 ± 8.773^bbb^

The results are expressed as mean ± standard error of the mean (SEM). Probability values of  ^aaaa^ *P* < 0.0001, ^aaa^ *P* < 0.001, ^aa^ *P* < 0.01, and ^a^ *P* < 0.05 were compared with normal control. Probability values of  ^bbbb^ *P* < 0.0001, ^bbb^ *P* < 0.001, ^bb^ *P* < 0.01, and ^b^ *P* < 0.05 were compared with disease control.

**Table 2:** Effect of ethyl acetate fraction and subfractions of flowers of *Pterospermum acerifolium* (L.) Willd on changes in blood glucose level.

Treatment	Blood glucose level (mg/dL)			
	Day 0	Day 14	Day 21	Day 28
Normal control	68.83 ± 1.014	75.33 ± 0.494	74.50 ± 0.428	73.83 ± 1.4
Diabetic control	296.5 ± 9.454	351.2 ± 11.84^aaaa^	360.7 ± 6.075^aaaa^	405.7 ± 6.344^aaaa^
PAFEF1	286.2 ± 8.975	238.8 ± 6.983	207.8 ± 7.674	183.7 ± 4.240
PAFEF2	302.3 ± 11.12	234.8 ± 16.8	205.7 ± 13.95	176.5 ± 9.942
PAFE1-1	285.2 ± 6.858	226.8 ± 7.726	200.2 ± 4.658	178.5 ± 8.655
PAFE1-2	300.7 ± 10.08	236.5 ± 13.25	207.3 ± 11.07	181.8 ± 8.623
PAFE2-1	282.8 ± 12.74	225 ± 8.629	180.5 ± 5.596^b^	151.8 ± 6.030^bb^
PAFE2-2	285 ± 7.904	193.5 ± 7.775^bb^	152 ± 6.698^bbb^	120.8 ± 5.375^bbb^
PAFE3-1	296.5 ± 10.30	270.7 ± 11.41^aa^	256.7 ± 10.74^aaa^	248.2 ± 10.99^aaa^
PAFE3-2	279.7 ± 11.26	265.3 ± 9.790^aa^	239 ± 12.40^aa^	229.5 ± 11.49^aaa^
STD drug	290.2 ± 7.115	178.5 ± 7.758^bbb^	134.5 ± 4.137^bbb^	106.5 ± 3.166^bbb^

The results are expressed as mean ± standard error of the mean (SEM). Probability values of  ^aaaa^ *P* < 0.0001, ^aaa^ *P* < 0.001, ^aa^ *P* < 0.01, and ^a^ *P* < 0.05 were compared with normal control. Probability values of  ^bbbb^ *P* < 0.0001, ^bbb^ *P* < 0.001, ^bb^ *P* < 0.01, and ^b^ *P* < 0.05 were compared with disease control.

**Table 3:** Effect of ethyl acetate fraction and subfractions of flowers of *Pterospermum acerifolium* (L.) Willd on body weight changes and glycosylated Hb.

Treatment	Body weight (gms)		Glycosylated Hb (%)
	Initial	Final	
Normal control	197.5 ± 2.707	241.1 ± 2.740	2.872 ± 0.277
Diabetic control	198.4 ± 4.845	149.7 ± 6.182	7.932 ± 0.109^aaaa^
PAFEF1	200.9 ± 3.605	205.2 ± 2.968	5.680 ± 0.060
PAFEF2	197.9 ± 5.206	210.7 ± 6.634	5.412 ± 0.023^b^
PAFE1-1	199.1 ± 4.202	201.3 ± 4.148	6.417 ± 0.067^aa^
PAFE1-2	199.9 ± 3.738	206.3 ± 3.983	5.892 ± 0.104
PAFE2-1	193.4 ± 2.815	201.8 ± 3.445	4.708 ± 0.055^bb^
PAFE2-2	199.9 ± 2.854	219.3 ± 3.157	4.227 ± 0.075^bbb^
PAFE3-1	203.7 ± 2.749	183.4 ± 3.410	6.770 ± 0.278^aa^
PAFE3-2	197.3 ± 2.953	184.4 ± 4.275	6.690 ± 0.049^aaa^
STD drug	203.2 ± 2.793	231.7 ± 3.740	3.337 ± 0.097^bbbb^

The results are expressed as mean ± standard error of the mean (SEM). Probability values of  ^aaaa^ *P* < 0.0001, ^aaa^ *P* < 0.001, ^aa^ *P* < 0.01, and ^a^ *P* < 0.05 were compared with normal control. Probability values of  ^bbbb^ *P* < 0.0001, ^bbb^ *P* < 0.001, ^bb^ *P* < 0.01, and ^b^ *P* < 0.05 were compared with disease control.

**Table 4:** Effect of ethyl acetate fraction and subfractions of flowers of *Pterospermum acerifolium* (L.) Willd on levels of serum liver enzymes.

Treatment	ALT (IU/L)	AST (IU/L)	ALP (IU/L)
Normal control	25.48 ± 0.337	32.14 ± 0.24	11.88 ± 0.24
Diabetic control	54.91 ± 0.45^aaaa^	72.35 ± 0.365^aaaa^	27.16 ± 0.041^aaa^
PAFEF1	44.07 ± 0.273	58.28 ± 0.348	22.39 ± 0.085
PAFEF2	41.60 ± 0.444	56.25 ± 0.387	21.33 ± 0.069
PAFE1-1	42.03 ± 0.711	53.48 ± 0.570	20.27 ± 0.064
PAFE1-2	40.94 ± 0.195	51.34 ± 0.364^b^	18.25 ± 0.054
PAFE2-1	38.75 ± 0.359^bb^	43.86 ± 0.770^bb^	17.21 ± 0.039
PAFE2-2	31.45 ± 0.371^bbb^	41.73 ± 0.484^bbb^	15.21 ± 0.053^b^
PAFE3-1	51.64 ± 0.478^aaaa^	69.76 ± 0.208^aaaa^	28.37 ± 0.073^aaaa^
PAFE3-2	49.77 ± 0.201^aaa^	68.90 ± 0.612^aaa^	27.32 ± 0.033^aaa^
STD drug	28.78 ± 0.251^bbbb^	37.17 ± 0.306^bbbb^	14.50 ± 0.064^bb^

The results are expressed as mean ± standard error of the mean (SEM). Probability values of  ^aaaa^ *P* < 0.0001, ^aaa^ *P* < 0.001, ^aa^ *P* < 0.01, and ^a^ *P* < 0.05 were compared with normal control. Probability values of  ^bbbb^ *P* < 0.0001, ^bbb^ *P* < 0.001, ^bb^ *P* < 0.01, and ^b^ *P* < 0.05 were compared with disease control.

**Table 5:** Effect of ethyl acetate fraction and subfractions of flowers of *Pterospermum acerifolium* (L.) Willd on levels of lipid parameter.

Treatment	Triglycerides (mg/dL)	Total cholesterol (mg/dL)	HDL-C (mg/dL)
Normal control	91.66 ± 0.495	57.53 ± 0.311	57.39 ± 0.407
Diabetic control	214.3 ± 1.261^aaaa^	159.2 ± 1.221^aaaa^	17.59 ± 0.514^aaaa^
PAFEF1	139 ± 1.289	105.2 ± 0.701	34.51 ± 0.671
PAFEF2	130.2 ± 1.488	97.90 ± 0.628	37.66 ± 0.458
PAFE1-1	125.2 ± 0.668	99.17 ± 0.708	38.53 ± 0.390
PAFE1-2	122 ± 0.917^bb^	94.43 ± 1.144	43.78 ± 1.178^b^
PAFE2-1	104 ± 1.234^bbb^	70.12 ± 0.812^bb^	50.69 ± 0.466^bb^
PAFE2-2	94.86 ± 1.075^bbbb^	66.36 ± 0.71^bbb^	54.41 ± 0.413^bbb^
PAFE3-1	184.8 ± 1.188^aaaa^	144 ± 0.648^aaaa^	21.60 ± 0.305^aaaa^
PAFE3-2	188 ± 0.816^aaa^	140 ± 0.546^aaa^	24.53 ± 0.501^aaa^
STD drug	125.8 ± 1.079	65.04 ± 0.731^bbb^	52.47 ± 0.315

The results are expressed as mean ± standard error of the mean (SEM). Probability values of  ^aaaa^ *P* < 0.0001, ^aaa^ *P* < 0.001, ^aa^ *P* < 0.01, and ^a^ *P* < 0.05 were compared with normal control. Probability values of  ^bbbb^ *P* < 0.0001, ^bbb^ *P* < 0.001, ^bb^ *P* < 0.01, and ^b^ *P* < 0.05 were compared with disease control.

**Table 6:** Effect of ethyl acetate fraction and subfractions of flowers of *Pterospermum acerifolium* (L.) Willd on levels of oxidative stress marker (TBARs) and antioxidant enzymes (Catalase, Glutathione, and SOD).

Treatment	TBARS	Catalase	Glutathione	SOD
Normal control	0.98 ± 0.16	74.82 ± 3.67	48.72 ± 2.21	7.86 ± 1.09
Diabetic control	1.74 ± 0.81^aaaa^	30.78 ± 3.16^aaaa^	23.46 ± 2.60^aaaa^	3.7 ± 0.34^aaaa^
PAFEF1	1.43 ± 0.62^a^	55.71 ± 3.22	36.86 ± 1.23^a^	4.50 ± 0.18^a^
PAFEF2	1.38 ± 0.86	57.82 ± 1.16	39.33 ± 2.71	4.88 ± 0.61
PAFE1-1	1.36 ± 0.16	54.17 ± 3.17	37.41 ± 1.81	5.19 ± 0.11
PAFE1-2	1.31 ± 0.32	59.23 ± 1.72	39.41 ± 2.86	5.43 ± 0.19
PAFE2-1	1.15 ± 0.16^b^	62.81 ± 1.81^b^	41.96 ± 1.33^bb^	6.10 ± 0.22^b^
PAFE2-2	1.10 ± 0.21^bb^	65.88 ± 1.26^bb^	43.83 ± 1.81^bb^	6.35 ± 0.13^bb^
PAFE3-1	1.71 ± 0.10^aaaa^	29.16 ± 2.33^aaa^	26.16 ± 1.33^aaa^	3.71 ± 0.31^aaa^
PAFE3-2	1.67 ± 0.23^aaa^	34.62 ± 1.70^aa^	28.33 ± 1.96^aaa^	3.96 ± 0.33^aaa^
STD drug	1.03 ± 0.50^bb^	68.92 ± 1.86^bbb^	44.67 ± 2.16^bbb^	6.41 ± 0.16^bbb^

Thiobarbituricacid reactive substances (TBARS) expressed as nM of MDA/mg protein; Catalase expressed as nmoles of H_2_O_2_ consumed/min/mg protein; Superoxide dismutase (SOD) expressed as U/mg protein; Glutathione expressed as *µ*g of GSH/mg protein. The results are expressed as mean ± standard error of the mean (SEM). Probability values of  ^aaaa^ *P* < 0.0001, ^aaa^ *P* < 0.001, ^aa^ *P* < 0.01, and ^a^ *P* < 0.05 were compared with normal control. Probability values of  ^bbbb^ *P* < 0.0001, ^bbb^ *P* < 0.001, ^bb^ *P* < 0.01, and ^b^ *P* < 0.05 were compared with disease control.

**Table 7:** HPLC chromatogram profile of standard reference compounds and fingerprint of PAFE2.

S. Number	Reference compounds	Retention time (Rt)	
		Standard mixture	PAFE2
1	Gallic acid	6.416	6.405
2	Chlorogenic acid	10.573	10.588
3	Rutin	12.191	12.204
4	Quercetin	18.497	18.197
5	Apigenin	20.173	19.924
6	Kaempferol	20.671	20.686
